# Pre-service knowledge, perception, and use of emergency contraception among future healthcare providers in northern Ghana

**DOI:** 10.1186/s40834-018-0082-9

**Published:** 2019-01-24

**Authors:** Shamsudeen Mohammed, Abdul-Malik Abdulai, Osman Abu Iddrisu

**Affiliations:** 1 Department of Nursing, College of Nursing and Midwifery, Nalerigu, Ghana; 2 Nurses’ and Midwives’ Training College, Tamale, Ghana

**Keywords:** Emergency contraception, Emergency contraceptive pills, Knowledge, Use, Perception, Nurses, Midwives, Tamale, Ghana

## Abstract

**Background:**

Emergency contraception, if used properly, can prevent up to over 95 % of unwanted and mistimed pregnancies. However, a number of obstacle including healthcare providers knowledge, perception, and attitude towards emergency contraception (EC) prevent women and adolescents from having access to EC.

**Methods:**

This was a cross-sectional study among 191 female final year nursing and midwifery students of Tamale Nurses and Midwives Training College in the Northern Region of Ghana. Purposive sampling method was used to sample 100 students from the nursing programme and 91 from the midwifery programme. Chi-square and Fisher’s exact tests were performed to determine factors associated with awareness about EC and use of EC.

**Results:**

Over four-fifths, 166(86.91%), of the participants indicated they had heard about EC prior to the study. Majority (80.10%) of the participants correctly indicated the time within which to take emergency contraceptive pills (ECPs). More than half, 105(54.97%), of the participants did not know the appropriate time within which to use IUD as EC. Almost four-fifths, 74(38.74%), of the participants indicated it is morally wrong to use EC and more than half, (*n* = 104, 54.45%), of them said EC use promotes promiscuity. Only 49(25.65%) participants said they had ever used ECP. Of the number that indicated ever-using ECP, 36(73.47%) cited condom breakage or slippage as the reason for using the method.

**Conclusion:**

Though there was a relatively high level of EC awareness and knowledge among the students, some students lacked detailed knowledge about the method, especially the use of IUD as EC. We found that it was easy to access EC in the study area but the use of EC was low among the students. Most of the students demonstrated a positive attitude towards EC, but many of them believed EC encourages promiscuous sexual behaviour and that it is morally wrong to use EC. The curriculum for nursing and midwifery education should provide opportunity for detailed information and practical knowledge on EC to demystify negative perceptions and attitudes of nursing and midwifery students towards EC and other forms of contraception and to improve their knowledge on EC.

## Background

Every woman and adolescent has the right to decide freely when to have children, how many, and with whom. These rights are upheld with emergency contraception (EC) in cases of unprotected intercourse, contraceptive failure, incorrect use of contraceptives, and coerced sex. Emergency contraception provides women and adolescents with a second opportunity to prevent an unplanned or mistimed pregnancy within three to five days of unprotected sexual intercourse by preventing or temporarily stopping ovulation or by causing a chemical change in sperm and egg before they meet [1]. It ensures women and girls are able to circumvent the socioeconomic and negative health outcomes of unplanned and unintended pregnancy [2]. Access to EC is particularly important for young women who are vulnerable to sexual abuse and often lack the skills and power to negotiate use of a condom [3].

According to the 2014 Ghana Demographic and Health Survey (GDHS), 30% of married women in Ghana had an unmet need for family planning services, with 17% having an unmet need for spacing and 13% having an unmet need for limiting. Largely, Ghanaian women had 0.6 children more than their ideal number of 3.6 children. This suggests that the total fertility rate (TFR) was 17% more than it would have been if unwanted births were avoided [4]. Unwanted pregnancies often lead to abortions performed in unsafe environments with complications such as haemorrhage, infections, infertility, or even death [5].

Emergency contraception, if used properly, can prevent up to over 95 % of unwanted and mistimed pregnancies. Copper-bearing intrauterine devices (IUDs) and emergency contraceptive pills (ECPs) are the two types of emergency contraception currently available for use. A Copper-bearing IUD is more than 99% effective in preventing pregnancy if inserted within 5 days of unprotected sexual intercourse. Emergency contraceptive pills include progesterone only pills and combined oral contraceptive pills and are more effective between 72 and 120 h of unprotected intercourse [1]. In Ghana, emergency contraceptive pills such as postinor-2, Lydia post pill, NorLevo, and pregnon are available in pharmacist, family planning clinics and can be procured without a medical prescription.

However, a number of obstacle including healthcare providers knowledge, perception, and attitude towards EC prevent women and adolescents from having access to EC [5]. Many women and adolescents are reluctant to purchase EC because of the negative attitude and perception of healthcare providers (nurses and midwives) about EC. Yet, since the inclusion of EC in the National Reproductive Health Service Policy and Standards by the ministry health in Ghana [6], limited studies have been conducted on the knowledge, attitude, and use of EC among female nursing and midwifery students in Northern Ghana. In view of that, this study was undertaken to assess the knowledge, awareness, attitude, and use of EC and to determine the association between these factors and the socio-demographic characteristics of female nursing and midwifery students of Tamale Nurses and Midwives Training College in Northern Ghana.

## Materials and methods

This was a cross-sectional study conducted at the Tamale Nurses and Midwives Training College in the Northern Region of Ghana. The college is one of the oldest health training institutions in the region. It currently runs three-year diploma programmes in nursing and midwifery. The study population consisted of all female student nurses and midwives of the college. Male students, health tutors, students who failed to give consent and those who were absent on the day data were collected were excluded. We included only female final year nursing and midwifery students who provided consent. The study protocol was reviewed and approved by the local research, quality assurance, and ethics committee of Tamale Nurses and Midwives Training College upon submission of a written permission from the principal of the college to the committee. All the participants were informed of the purpose of the study and their rights and role during the study. Participants indicated their consent to participate in the study by voluntary signing a consent form designed for the study. Confidentiality of the information collected was ensured.

### Sampling and data collection

The sample size for the study was calculated using Yamane’s formula [7] for proportions based on a population of 240 final year female nursing and midwifery students, under the assumption of 95% confidence interval, 5% margin of error, and 20% non-response rate. A sample size of 180 was estimated but was increased to 191 to broaden the scope of the study. The sample size was allocated to the nursing and midwifery programmes using proportion to size approach. Purposive sampling method was then used to sample 100 students from the nursing programme and 91 from the midwifery programme. The students were acquainted with the objective and purpose of the study and informed of their rights during the study. Data for the study were collected with a structured questionnaire on knowledge, perception, and use of emergency contraception. We also collected information on some socio-demographic characteristics (age, marital status, religion, and programme of study) of the students. The questionnaire was designed after considering variables that were included in similar studies. Two senior midwives and a public health professional reviewed the questionnaire for construct and content validity. Then the questionnaire was piloted on 30 first-year nursing and midwifery students to ascertain the clarity and practicability of the questions and to identify poorly constructed items and ambiguities that may be encountered during data collection. Suggested changes from the review and pilot study were made before the actual data collection. Three trained health tutors administered the questionnaire to the nursing and midwifery students in classrooms during school hours.

### Data analysis

Data were checked for completeness, entered into Microsoft Excel spreadsheet and analysed using Stata v14. Descriptive analyses were presented in tables as frequencies and percentages. Chi-square test was used to assess the association of knowledge about EC, use of EC, and socio-demographic characteristics of the participants. Socio-demographic characteristics with expected values less than 5 were analysed using Fisher’s exact test. The significant level for the test was set at p 0.05.

## Results

### Background characteristics of participants

A total of 191 female nursing and midwifery trainees participated in the study. Table 1 presents the description of background characteristics of the participants. More than half, 98(51.3%), of the participants were in the age range of 23–27 years while 15(7.8%) of them were in the age range of 33–37 years. Majority, 175(91.6%), of the participants were not married. Only 16(8.4%) participants indicated they were married at the time of the study. More than half of the participants were Christians (*n* = 106, 55.5%) and nursing students (*n* = 100, 52.4%).

**Table 1:** Background characteristics of participants

Characteristics	Number	percent
Age (years)		
18–22	78	40.84
23–27	98	51.31
33–37	15	7.85
Marital status		
Married	16	8.38
Single	175	91.62
Religion		
Islam	85	44.50
Christianity	106	55.50
Programme of study		
Nursing	100	52.36
Midwifery	91	47.64

### Participant’s awareness and knowledge about emergency contraception

Table 2 shows participants awareness and knowledge about emergency contraception. Over four-fifths, 166(86.9%), of the participants indicated they had heard about emergency contraception prior to the study. Eighty-nine (53.6%) of them had heard about it within 6 months to 3 years prior to the study. Only 34(20.5%) of them indicated they heard about it less than 6 months to the study. More than half of the participants who had heard about emergency contraception prior to the study cited health care providers (*n* = 88, 53.0%) as the source of information, followed by friends (*n* = 40, 24.1%), media (*n* = 26, 15.7%), and family (*n* = 12, 7.2%). Over four-fifths of the participants answered correctly that measures can be taken to prevent pregnancy after unprotected sex (*n* = 165, 86.4%) and that emergency contraception is used after unprotected sex to prevent unwanted pregnancy (*n* = 161, 84.3%). One hundred and fifty-three (80.1%) correctly indicated the time within which to take an emergency contraceptive pill. However, more than half, 105(54.9%), of the participants did not know the appropriate time within which to use IUD as an emergency contraceptive. Thirty (15.7%) participants said emergency contraception induces abortion.

**Table 2:** Participants awareness and knowledge about emergency contraception

Knowledge variables	Number	Percent
Ever heard of EC		
Yes	166	86.91
No	25	13.09
When did you first hear about EC (*n* = 166)		
Less than 6 months ago	34	20.48
6 months to 3 years ago	89	53.61
over 3 years ago	43	25.90
Source of information about EC (*n* = 166)		
Friends	40	24.10
Family members	12	7.23
Health care provider	88	53.01
Media	26	15.66
Measures can be taken to prevent pregnancy after unprotected sex		
Yes	165	86.39
No	11	5.76
Don’t Know	15	7.85
When is it appropriate to use EC		
After unprotected sex	161	84.29
To abort an unwanted pregnancy	6	3.14
As an ongoing contraceptive	9	4.71
Don’t Know	15	7.85
Time appropriate to use ECP		
Within 72–120 h of unprotected sex	153	80.10
More than 120 h of unprotected sex	17	8.90
Don’t Know	21	10.99
When is an IUD effective as an emergency contraceptive		
Only within 72 h of unprotected sex	70	36.65
Within 5 days of unprotected sex	16	8.38
Don’t Know	105	54.97
EC is an early method for abortion		
Yes	30	15.71
No	136	71.20
Don’t Know	25	13.09

As shown in Table 3, awareness about EC increased with advancement in age as 79.5, 91.8, and 93.3% of the participants in the age range of 18–22 years, 23–27 years, and 33–37 years indicated they had ever heard of EC, respectively. There was no statistically significant association between awareness about EC and marital status (*p* = 0.397), religion (*p* = 0.957), and programme of study (*p* = 0.211).

**Table 3:** Factors associated with awareness about emergency contraception among study participants

Variables	Ever heard of EC				X^2^	*P* value
	Yes		No			
	n	%	n	%		
Age (years)						
18–22	62	79.49	16	20.51		
23–27	90	91.84	8	8.16	6.4130	0.040^¶^
33–37	14	93.33	1	6.67		
Marital status						
Married	15	93.75	1	6.25	0.7180	0.397^¶^
Single	151	86.29	24	13.71		
Religion						
Islam	74	87.06	11	12.94	0.0029	0.957
Christianity	92	86.79	14	13.21		
Programme of study						
Nursing	84	84.00	16	16.00	1.5635	0.211
Midwifery	82	90.11	9	9.89		

^¶^Fisher’s exact test

### Participant’s perception and attitude towards emergency contraception

Participants perception and attitude towards emergency contraception was assessed and presented in Table 4. Almost two-fifths, 74(38.7%), of the participants indicated it is morally wrong to use EC and more than half, 104(54.5%), of them said EC use promotes promiscuity. One hundred and three (53.9%) and 158(82.7%) participants agreed unmarried adolescents could use EC and that correct use of EC is safe, respectively. Nonetheless, most of them (*n* = 92, 48.2%) did not want EC to be made widely available for use. Fifty-five (28.8%) participants incorrectly believed EC induces abortion and 125 (65.5%) them said it was easy to access EC in the study area.

**Table 4:** Participants perception and attitude towards emergency contraception

Variables	Agree		Disagree		Not Sure	
	No.	%	No.	%	No.	%
It is morally wrong to use EC	74	38.74	85	44.50	32	16.75
EC encourages promiscuity	104	54.45	73	38.22	14	7.33
Unmarried adolescents can use EC	103	53.93	72	37.70	16	8.38
Correct use of EC is safe	158	82.72	22	11.52	11	5.76
EC should be widely available	81	42.41	92	48.17	18	9.42
EC is one way of abortion	55	28.80	105	54.97	31	16.23
It is easy to procure EC	125	65.45	53	27.75	13	6.81

### Use of emergency contraceptive pill

Table 5 illustrates participants utilization of emergency contraceptive pills (ECPs). More than half, 110(57.6%), of the participants indicated they had never used ECP. Only 49(25.7%) of them said they had ever used ECP. The remaining 16.8% were not sure or could not remember whether they had ever used an ECP. Of the number that indicated ever using ECP, 36(73.5%) cited condom breakage or slippage as the reason for using the method, 41(83.7%) mentioned pharmacy/chemical shop as the major source of EC commodities, and 35(71.4%) said they had no difficulty obtaining ECP. Eighty-three (58.5%) of the participants that indicated they had never used ECP or could not remember ever using it, said they will use the pill in the event of unprotected sex to prevent unwanted pregnancy. More than half, 101(52.9%), of the participants said they will recommend ECP to others for the prevention of unwanted pregnancy.

**Table 5:** Emergency contraceptive pill utilization among study participants

Variables	Number	Percent
Ever use emergency contraceptive pill		
Yes	49	25.65
No	110	57.59
Not sure/can’t remember	32	16.75
Reason for using EC (*n* = 49)		
Condom breakage or slippage	36	73.47
Missed pill	11	22.45
Forced sex	2	4.08
Source of used EC commodities (*n* = 49)		
Pharmacy/chemical shop	41	83.67
Health facility/healthcare provider	5	10.20
Friends	3	6.12
Did you experience any difficulty obtaining EC (*n* = 49)		
Yes	14	28.57
No	35	71.43
Would you use EC to prevent unwanted pregnancy in the event of unprotected sex (*n* = 142)		
Yes	83	58.45
No	59	41.55
Would you recommend EC to prevent pregnancy		
Yes	101	52.88
No	46	24.08
Not sure	44	23.04

As shown in Table 6, the use of ECP was significantly associated with the age of participants (*p* = 0.010), and their religion (*p* = 0.042). The perception that it is easy to obtain EC (p = < 0.001), and that the correct use of EC is safe (*p* = 0.006) were also significantly associated with the use of EC. However participants programme of study (*p* = 0.799), marital status (*p* = 1.000), and the perception that EC promotes promiscuity (*p* = 0.639), did not show a statistically significant association with the use of ECP.

**Table 6:** Factors associated with utilization of emergency contraceptive pill

Variables	Ever used ECP (*n* = 159)				X^2^	*P* value
	Yes		No			
	n	%	n	%		
Age (years)						
18–22	19	29.69	45	70.31		
23–27	22	26.19	62	73.81	9.9439	0.010^**¶**^
33–37	8	72.73	3	27.27		
Marital status						
Married	4	33.33	8	66.67	0.0385	1.000^**¶**^
Single	45	30.61	102	69.39		
Religion						
Islam	16	22.54	55	77.46	4.1275	0.042
Christianity	33	37.50	55	62.50		
Programme of study						
Nursing	27	30.00	63	70.00	0.0650	0.799
Midwifery	22	31.88	47	68.12		
It is easy to procure EC						
Agree	45	43.27	59	56.73		
Disagree	4	8.70	42	91.30	22.1307	< 0.001^**¶**^
Not sure	0	0.00	9	100.00		
Correct use of EC is safe						
Agree	48	35.56	87	64.44		
Disagree	1	6.25	15	93.75	9.5146	0.006^**¶**^
Not sure	0	0.00	8	100.00		
EC encourages promiscuity						
Agree	26	30.23	60	69.77		
Disagree	22	33.33	44	66.67	1.1070	0.639^**¶**^
Not sure	1	14.29	6	85.71		

^¶^Fisher’s exact test

## Discussion

It is evident from our findings that there is a high level of awareness of EC among the participants. This is comparable to findings of several studies [8–11]. Further, the students had good knowledge about emergency contraception. This is understandable considering the fact that family planning is a major part of the curriculum for both the nursing and midwifery programmes. A higher knowledge about EC will build up their capacity to provide accurate and effective information on EC to prevent unplanned and unwanted pregnancies. Similar studies in Korea and America found a significantly higher knowledge of ECPs among participants who had received education and formal content on EC [12, 13]. Despite the good knowledge, more than half of the students did not know IUD could prevent unwanted pregnancy beyond 72 h of unprotected sexual intercourse, which is consistent with a previous study among nurse practitioner students [13]. This is worrying, given that women seeking to use IUD depend on healthcare providers for information. Lack of knowledge of the correct period to use IUD may affect the accuracy of information provided by these future nurses and midwives to women seeking to use IUD as EC and could lead to underutilisation of the method. This underscores the need to broaden and provide detail education on family planning, especially EC, in Nursing and Midwifery Training Schools.

It was disturbing to find that the student nurses and midwives believed EC use encourages casual and indiscriminate sexual intercourse, which contradicts the findings of Sorhaindo et al. [14] but confirms those of Celik et al. [11]. Available literature does not support the argument that EC use promotes unprotected sexual intercourse or discourage use of other methods of contraception. This highlights the need to intensify education campaigns targeted at demystifying negative perceptions and attitudes towards EC and other forms of contraception. More of a concern is the fact that about one-third of the students incorrectly believed it is morally wrong to use EC. These findings are worrying given that the participants were final year students who will soon graduate to provide healthcare services including family planning and contraception services to women and adolescents. The reported perception may possibly affect their attitude towards the delivery of information about EC and counselling of women seeking to use EC and perhaps other methods of contraception. However, when Delaram and Rafie [9] surveyed medical students in Iran they found that the majority of the students did not agree it was unethical to use EC and 43% of them disagreed that EC use will lead to irresponsible sexual behaviour [13]. Furthermore, Celik et al. reported 61% disagreement with the perception that ECPs use is unethical when they surveyed nursing students in Turkey [11]. The varying findings may be due to the difference in study settings as the Iranian and Turkish studies were conducted among university students who may have received a higher content on family planning compared with the college students in our study.

All women and girls at risk of an unintended pregnancy have a right to access emergency contraception. Furthermore, evidence suggests that ECPs use is safe and the side effects associated with its use are similar to those of oral contraceptive pills, and will normally resolve without further medications [1]. In the current study, the majority of the students agreed that correct use of EC is safe and that it is easy to access EC in the study area. This suggests that the students could easily procure EC to prevent an unplanned pregnancy in case of unprotected sexual intercourse. In line with our findings, Kang and Moneyham reported easy accessibility of EC as the highest scored attitude of college students towards EC in America [12].

EC disrupts ovulation and reduces the likelihood of pregnancy. It cannot prevent implantation of a fertilized egg, harm a developing embryo, or end a pregnancy (UNFPA 2013). Nonetheless, a little over one-fourth (28.8%) of the students in this study wrongly agreed EC is an abortifacient which agrees well with the findings of Lee et al. [13] in America but higher than those of Delaram and Rafie [9] in Iran. In the American study, 27% of the sample believed EC have a similar mechanism of action as mifepristone (an abortifacient), and 10% inaccurately believed EC interrupts pregnancy [13]. This misconception needs to be corrected if we are to reduce the rate of unwanted and mistimed pregnancies because nurses and midwives with this perception are less likely to accurately advise and educate women seeking to use EC.

There is no age limit or absolute medical contraindications to the use of EC (WHO 2018). Access to EC is especially important for adolescents who often lack the skills or power to negotiate condom use and are vulnerable to sexual exploitation [3]. In the current study, more than half of the students agreed that unmarried adolescents could use EC. Our finding contradicts what was reported by Celik et al. [11]. Generally, use of EC was low among the participants in this study. This suggests that the participants either use other forms of contraception or are not engaged in unprotected sexual intercourse since only 8% of them were married. Most of those who indicated they had ever used EC cited condom breakage or slippage as the reason for using EC, which is comparable to what was found in Jamaica [14]. This highlights the need for further education on how to properly use and dispose of condoms.

## Conclusion

Though there was a relatively high level of EC awareness and knowledge among the students, some students lacked in-depth knowledge about the method, especially the use of IUD. We found that it was easy to access EC in the study area but the use of EC was low among the students. Most of the students demonstrated a positive attitude towards EC, but many of them believed EC encourages promiscuous sexual behaviour and that it is morally wrong to use EC. The curriculum for nursing and midwifery education should provide opportunity for detailed information and practical knowledge on EC to demystify negative perceptions and attitudes of nursing and midwifery students towards EC and other forms of contraception and to improve their knowledge on EC.

## Abbreviations

EC: Emergency contraception
ECP: Emergency contraceptive pills
IUD: Intrauterine device
